# Growing Trend of Alternative Tobacco Use Among the Nation’s Youth: A New Generation of Addicts

**DOI:** 10.5811/westjem.2016.1.29383

**Published:** 2016-03-02

**Authors:** John R. Marshall, Shahram Lotfipour, Bharath Chakravarthy

**Affiliations:** University of California, Irvine, Department of Emergency Medicine, Orange, California

## Abstract

The Centers for Disease Control and Prevention (CDC) has published significant data and trends related to the rising epidemic of usage of alternate forms of tobacco among the nation’s youth. For the first time ever, the use of the electronic cigarette (e-cigarrette) has surpassed traditional cigarette usage in adolescents. E-cigarettes are battery-operated products designed to deliver aerosolized nicotine and other flavors to the consumer. Most look like conventional cigarettes but some resemble everyday items such as pens, USB drives, and memory sticks.^1^ In the following article, we present findings from the CDC’s Morbidity and Mortality Weekly Report with commentary on the state of this growing epidemic and barriers to effective screening methods.

## CDC MMWR Findings

In the April 17,2015 issue of the Morbidity and Mortality Weekly Report (MMWR), the Centers for Disease Control and Prevention (CDC) and Food and Drug Administration (FDA) published data and trends concerning tobacco use among middle (grade 6–8) and high school students (grade 9–12). The report clearly showed that between 2011 and 2014, statistically significant increases were observed among students for the use of electronic cigarettes and hookahs that has offset the overall decrease in more traditional tobacco products such as cigarettes and cigars.

To determine the prevalence and trends of past 30-day use of nine tobacco products (cigarettes, cigars, smokeless tobacco, e-cigarettes, hookahs, tobacco pipes, snus, dissolvable tobacco, and bidis), the CDC and FDA analyzed data from the 2011–2014 National Youth Tobacco Surveys (NYTS). The NYTS is a cross-sectional, school-based, self-administered, written questionnaire administered to students to monitor the impact of comprehensive tobacco control polices and strategies and inform the FDA’s regulatory actions. Participants were asked about current usage of any tobacco products. “Current usage” was defined as using a tobacco containing product ≥1 time during the past 30 days. Tobacco use was defined as “any tobacco containing product,” and “≥2 tobacco product usage” was defined as use of two or more tobacco products. Orthogonal polynomials were used with logistic regression analysis to test for significant linear and non-linear trends.

The CDC found that in 2014, 24.6% of high school students reported current use of a tobacco-containing product with 12.7% reporting using ≥2 tobacco products. Of all types, e-cigarettes (13.4%) were the most common tobacco products used while only 9.2% used traditional cigarettes. From 2011 to 2014, statistically significant non-linear increases were observed among high school students for current e-cigarette usage (1.5 to >13.4%) while statistically significant linear decreases were observed for current cigarette usage (15.8 to >9.2%) and cigar usage (11.6 to 8.2%). Current usage of any tobacco and ≥2 tobacco products from 2011–2014 did not change significantly and was (24.2 to >24.6%) and (12.5 to >12.7%) respectively. Overall in 2014, of the 4.6 million total middle and high school students using any tobacco product, 2.4 million used e-cigarettes and 1.6 million used hookah. Since 2013, e-cigarette usage has tripled from 4.5% (660,000) to 13.4% (two million) in high school students from 2013–2014, and from 1.1% (120,000) to 3.9% (450,000) in middle school students from 2013–2014. Significant increases were also observed for hookah usage for high school and middle school students from 2013–2014.

Overall, the CDC found statistically significant decreases in in the use of cigarettes, tobacco pipes, bidis, cigars, and snus. Compounded with the massive increase in usage of e-cigarettes and hookah, this has offset the use of other tobacco products, resulting in no change to the overall current tobacco use among middle and high school students. This was the first time in NYTS that e-cigarette usage surpassed the usage of cigarettes.

The CDC acknowledged three limitations in this report. Data were only collected from youth in attendance in either public or private schools. Secondly, missing responses in the report were included as “non-usage.” Lastly, it was believed that the wording and placement questions about the use of e-cigarettes, hookahs, and tobacco pipes may have had an impact on the reported use of these products.^2^

## Commentary

It’s 8pm and you’ve smoothly made it through half of your intake shift. The triage nurse brings in your next patient, a 16-year-old male with a complaint of “cough” for three days. He divulges to you a history of mild intermittent asthma and you begin your treatment regimen. Later on your re-evaluation, you find he is feeling much better, his peak flows have improved, and you begin your discharge process. Your electronic medical record prompts you to inquire about tobacco usage, and, expecting a negative answer, you comply. To your astonishment your patient admits to smoking. To your relief you find that his mother is aware of this and they both understand that this is contributing to his worsening asthma. They tell you they are trying to figure out the best way for him to quit and they have even been considering the “e-cigarette” because an aunt told them it was a healthier alternative. You pause and consider your options. You have referred countless adults for tobacco intervention, but never a child, and really how bad are these e-cigarettes anyway?

Emergency physicians (EP) throughout the nation, regardless of location, share the burden of screening for tobacco use in their patients and referring them accordingly. The MMWR is useful in identifying a new trend in the use of non-conventional tobacco products such as e-cigarettes affecting our nation’s youth, but provides little insight into clinical significance at the provider level when dealing with adolescents. With up to 46 million Americans using the emergency department (ED) as their primary form of care, the additional volume of patients poses an increased responsibility to address key health issues without sacrificing efficiency,^3^

Each day, over 3,800 adolescents between 12–17 smoke their first cigarette, contributing to the 1.5 million youth who begin to smoke each year,^4^,^5^ Research establishing the effectiveness of prevention and cessation interventions in adolescents is necessary to minimalize immediate adverse health effects in young smokers, such as asthma, decreased lung function, and early atherosclerosis.^6^,^7^ It is estimated that a 26% decrease in adolescent smoking prevalence would result in an annual savings of 100,000 lives and 1.6 million years of human life.^8^

In adults, tobacco screening methods such as the “5 As,” (ask, advise, assess, assist, and arrange for treatment) and many well-studied cessation options such as nicotine replacement therapy (NRT), telephone counseling, and FDA-approved psychotropic medications are available for the provider to use at his or her discretion.^9^ In adolescents, however, data on methods for screening and intervention are available, but have been far less studied than in adults. The “Surgeon General’s Report on Preventing Tobacco Use Among Youth and Young Adults” concluded there is robust evidence for effective strategies for reducing tobacco use among adolescents. This includes efforts of legislature (i.e. taxation, clean air policies, regulations on youth access, advertising bans, product labeling), large community environments (mass media campaigns, community interventions, state level tobacco control programs), and small social environments (clinical settings, school, family, youth programs) where the EP fits in. The Surgeon General concluded that it was the combined global effort from the above sectors that was effective in reducing the initiation, prevalence, and intensity of smoking among youth and young adults.^7^

The 2009 NYTS suggested that physicians as a whole could improve when it comes to screening for tobacco usage in adolescents. These data found that only 21% of adolescents recalled a physician asking them if they smoked in the last 12 months with only 7% of physicians suggesting to stop smoking.^10^ One possible reason for reluctance to screen at the provider level could be concern over financial incentive or lack of training. In 2010, the U.S. Department of Health changed the Medicare-approved reimbursement for tobacco cessation counseling to include all tobacco users for sessions longer than three minutes.^11^ With the current implementation of the ICD-10, codes for “Nicotine Dependence, other tobacco products” for both asymptomatic and symptomatic patients are now included as billable diagnoses.^12^ To assist the provider in addressing tobacco cessation in adolescent patients, in 2008 a Public Health Service (PHS)-sponsored update to the Treating Tobacco Use and Dependence Clinical Practice Guideline was published, which reviewed the effectiveness of tobacco use interventions for adolescent smokers and made the following recommendations:

- Recommendation 1: Clinicians should ask pediatric and adolescent patients about tobacco use and provide a strong message regarding the importance of totally abstaining from tobacco use (Strength of Evidence=C).
- Recommendation 2: Counseling has been shown to be effective in treatment of adolescent smokers. Therefore, adolescent smokers should be provided with counseling interventions to aid them in quitting smoking (Strength of Evidence=B).
- Recommendation 3: Secondhand smoke is harmful to children. Cessation counseling delivered in pediatric settings has been shown to be effective in increasing cessation among parents who smoke. Therefore, to protect children from secondhand smoke, clinicians should ask parents about tobacco use and offer them cessation advice and assistance (Strength of Evidence=B).^13^

The PHS also stated that regarding pharmacologic therapies, such as NRT and psychotropic medications, due to lack of evidence on effectiveness on promoting long-term smoking abstinence, prescription of these products is not recommended in people younger than 18 years old.^13^,^14^

Currently, we are seeing a rising trend in the use of non-conventional tobacco products with the e-cigarette surpassing traditional cigarette usage in adolescents for the first time ever. Immune from FDA regulation, these products are not subject to restrictions on marketing, manufacturing, labeling, and distribution. Few case studies have even reported incidences of mechanical bodily injury occurring due to malfunctioning e-cigarettes batteries.^15^ Also, without widely accessible information on adverse health risks come incorrect public perceptions on safety. One study found that although adolescents were familiar with the health risks of traditional cigarettes, they were unsure of any associated with e-cigarettes.^16^

Just recently, in 2014 the FDA proposed a rule to extend its control to deem all tobacco-containing products subject to FDA jurisdiction. With this proposal, non-conventional products such as the e-cigarette would be subject to the same provisions as traditional cigarettes in areas such as advertising and ingredient disclosure.^17^ If this happens, these products could then be subject to extensive scientific review unmasking harmful health risks.

Although the success of achieving tobacco cessation in our nation’s adolescents remains a global effort, EPs can play a vital part simply by asking, advising, and referring their patients to counseling. With the recent MMWR identifying the growing trend in usage of non-conventional tobacco products in our nation’s youth, EPs should take on the onus to include screening for non-conventional tobacco products, as well as familiarize themselves with appropriate referrals for counseling.
